# Impact of Dysmenorrhea on Academic Performance Among Haramaya University Undergraduate Regular Students, Eastern Ethiopia

**DOI:** 10.3389/frph.2022.939035

**Published:** 2022-07-06

**Authors:** Tiruye Tilahun Mesele, Hiwotie Getaneh Ayalew, Asmra Tesfahun Syoum, Tazeb Alemu Antehneh

**Affiliations:** ^1^Department of Midwifery, School of Midwifery, College of Medicine and Health Science, University of Gondar, Gondar, Ethiopia; ^2^Department of Midwifery, School of Nursing and Midwifery, College of Medicine and Health Science, Wollo University, Dessie, Ethiopia

**Keywords:** dysmenorrhea, Ethiopia, academic performance, absenteeism, impact

## Abstract

**Background:**

Dysmenorrhea is the most common gynecological problem among students. The disease affects students' academic performance, although studies carried out in Ethiopia primarily focused on the prevalence aspects rather than the impacts of dysmenorrhea on academic performance and its associated factors. Therefore, this study focused on the prevalence of the impact of dysmenorrhea on academic performance and its associated factors among undergraduate female students of Haramaya University in eastern Ethiopia.

**Methods:**

A cross-sectional study design was conducted from February to March 2020. A multistage random sampling technique was applied and a total of 356 students were included in the study. To select students, simple random sampling was used and the sample size was proportionally allocated with respect to the total number of each selected department. A semi-structured and a pre-tested self-administered questionnaire were used. The descriptive result was presented as a proportion whereas the analytic part was presented with an adjusted odds ratio.

**Result:**

The prevalence of the impact of dysmenorrhea on academic performance was 266 [(74.7%):95%CI (70.0, 79.5%)]. Premenstrual syndrome [AOR = 4.86:95%CI (2.13, 11.06)], early menarche [AOR = 4.89:95%CI (2.03, 11.77)], moderate/severe dysmenorrhea pain intensity [AOR = 8.53:95%CI (4.45, 16.39)], and students monthly pocket money <150ETB [AOR = 3.91:95%CI (1.48, 10.29)] were significantly associated with the occurrence of the impact of dysmenorrhea on academic performance. The most common impacts were difficulty in studying followed by loss of concentration in the class.

**Conclusion and Recommendation:**

There was a high prevalence of impact of dysmenorrhea on academic performance among undergraduate female students of the Haramaya University. Awareness should be created among Haramaya university authorities and teachers about the academic performance impact of premenstrual syndrome and dysmenorrhea pain intensity to provide psychological and academic guidance, and managing mechanisms for the affected students. Haramaya University should also establish medical care for the affected students.

## Background

Dysmenorrhea is painful menstruation which is a common gynecologic problem that significantly affects the routine life and academic performance of most of the affected female students (1–3). Dysmenorrhea is often accompanied by sweating, tachycardia, headache, nausea, vomiting, diarrhea, breast tenderness, and mood changes, which were the most common causes of class absenteeism and difficulty in studying (4–6).

Dysmenorrhea is the most common gynecological problem which has an impact on most of the students in their academic performance and daily activities (1, 3, 6–8). Globally, the prevalence of dysmenorrhea ranges from 16 to 91%, and 10–20% of them suffer from severe dysmenorrhea, which is the leading cause of recurrent school absenteeism (80%), loss of class concentration (66.8%), no active participation (47.4%), inability do homework (21%), fail in an exam (15.4%), and limited activity (29.9%) (5, 6, 9–13). The overall prevalence of impact of dysmenorrhea on academic performance in Ethiopia ranges from 20 to 88.3% (5, 6, 10).

The major associated factors with dysmenorrhea are early menarche, age <20, family history of dysmenorrhea, premenstrual syndrome, anxiety, smoking, and lack of physical exercise (5, 6, 9, 11, 13–15). However, studies carried out in Ethiopia (16) primarily focused on the prevalence aspects rather than the impacts of dysmenorrhea on academic performance and associated factors. Therefore, this study focused on the prevalence of the impact of dysmenorrhea on academic performance and its associated factors among undergraduate female students of Haramaya University in eastern Ethiopia.

## Materials and Methods

### Study Period, Area, and Design

The study was conducted from February 24 to March 24 /2020 at the Haramaya University. It is one of the oldest public universities in Ethiopia which was established in 1954. The university is located between Dire Dawa and Harar town, 510 kilometers away from the east of Addis Ababa. A university-based cross-sectional study design was applied.

All the female undergraduate regular students at Haramaya University were the source population during the study period, whereas all the female undergraduate regular students in selected departments of the three colleges at Haramaya University during the data collection period were the study population.

Any regular undergraduate dysmenorrheic female student in the selected departments had the chance to be included in this study whereas students who were ill and could not give a response during the data collection period were excluded from this study.

### Sample Size and Sampling Procedure

In this study, 356 female Haramaya university dysmenorrheic undergraduate students participated.

A multistage sampling technique was used. From 11 colleges and one school of directorate, which coordinates only freshman students of Haramaya University and is considered as one college for this study, 3 colleges were selected namely: College of Social Sciences and Humanities, College of Natural and Computational Sciences, and College of Health and Medical Science were selected using simple random sampling methods. From these 3 colleges, 13 departments were selected based on the number of departments in each college. The total sample size was proportionally allocated based on the total female students in each selected college and department and stratified by the year of study. Finally, the study participants were selected by simple random sampling.

### Data Collection Tools, Procedures, and Quality Assurance

Data collection questionnaires were an adaptation from previous similar studies (6, 9, 16–22). Data was collected by using a pre-tested and self-administered questionnaire design in English. The tool includes four sections, such as socio-demographic characteristics of students, the menstrual pattern of students, dysmenorrhea characteristics of students, and impacts of dysmenorrhea on students' academic performance. The data were collected by a 6-degree midwife clinical staff and supervised by 2 master midwives. Data collectors have explained the aims of this study to the students and taken consent from students and then continued it. The questionnaire was pre-tested by Dire Dawa university students. One day of training for data collectors and supervisors was given to understand the objective of the study, methods of data collection, handling of data, and ways of approaching the students. The supervisors were checking the activities of each data collector and all the filled questionnaires for their completion, clarity, and proper identification of the students.

### Operational Definitions

A student was considered to have dysmenorrhea if she said “Yes” about menstrual pain and had one or more of the following complaints: abdominal pain, groin pain, pelvic pain, back pain, or thigh pain before and/or during her menstrual periods (22).

Impact on academic performance: students said” Yes” about menstrual pain affects academic performance (6, 10, 12).

### Methods of Data Processing and Analysis

After data collection, data were cleaned and coded before data entry. After cleaning and coding, it was to be entered into Epi-Data version 3.1 and exported to STATA 14 for analysis. A descriptive summary was used to describe the characteristics of students in terms of frequencies and proportion and then the information was presented by text and tables.

Both bivariate and multivariable analysis logistic regression models were used to identify factors associated with the outcome variable. Those variables in a bivariate analysis whose *p* < 0.25 were taken as a candidate for multivariable analysis. Multi-collinearity was checked and model fitness was tested by the Hosmer Lemeshow test. Finally, variables whose *p* < 0.05 in the multivariable analysis were considered as a statistically significant association and the result was reported as the adjusted odds ratio.

## Results

### Socio-Demographic Characteristics of Students

Students' age ranges from 18 to 26 years. The majority, 237 (66.57%), of the students were categorized under the age group of 21–24 years. Most of the students, 242 (67.98%), were in the 3^rd^ year and above. The majority of the students, 211 (59.27%), were in the College of Health and Medical Science and 202 (56.74%) had more than 300ETB monthly pocket money. Half, 226 (63.48%), of the student's mothers were not formally educated. Most of the students, 276 (77.53%), did not participate in physical exercise. The majority of the students, 274 (76.97.5%), had a history of anxiety (Table 1).

**Table 1:** Socio-demographic characteristics of female undergraduate students, at Haramaya University, Ethiopia, 2020.

**Variables**		**Frequency**	**Percent**
Age of the students (in years) (*n* = 356)	18–20 21–24 ≥25	101 237 18	28.37 66.57 5.06
Monthly pocket money of the student's (*n* = 356)	<150 ETB 150–300ETB >300 ETB	69 85 202	19.38 23.88 56.74
The academic year of the students (*n* = 356)	2^nd^ year 3^rd^ year and above	114 242	32.02 67.98
College of the students (*n* = 356)	CHMS CSSH CNCS	211 78 18.82	59.27 21.91 18.82
Educational status of student's mother (*n* = 356)	No formal education Formal education	226 130	63.48 36.52
Doing physical exercise (*n* = 356)	Yes No	80 276	22.47 77.53
History of anxiety (*n* = 356)	Yes No	274 82	76.97 23.03

### The Menstrual Pattern of Students

About 156 (43.82%) students experienced menarche at the age of 13–14 years. About 168 (47.19%) students had a regular menstrual cycle and most of the students, 165 (46.35%), hade menses within 21–35 days intervals. About 192 (53.93%) students had a duration of menstrual flow of fewer than 3 days and 217 (60.96%) had used less than three pads per day (Table 2).

**Table 2:** Reproductive characteristics and menstrual pattern of female undergraduate students, at Haramaya University, Ethiopia, 2020.

**Variables**		**Frequency**	**Percent**
Age at menarche (*n* = 356)	≤ 12 years 13–14 years ≥15 years	125 156 75	35.11 43.82 21.07
History of sexual intercourse (*n* = 356)	Yes No	20 336	5.62 94.38
Menstrual regularity (*n* = 356)	Yes No	168 188	47.19 52.81
Menstrual interval in days (*n* = 356)	<21 days 21–35 days >35 days	156 165 35	43.82 46.35 9.83
Duration of menstrual flow (*n* = 356)	<3 days 3–7 days >7 days	192 124 40	53.93 34.83 11.24
Number of pads changed per day (*n=* 3 56)	<3 pads 3–7 pads >7 pads	217 126 13	60.96 35.39 3.65

### Prevalence of Dysmenorrhea Pain Intensity

Dysmenorrhea that hindered academic performance and required medication during each menstrual cycle was classified as moderate to severe in 245 (68.8%) and mild pain in 111 (31.2%). Most of the students, 239 (67.1%), had dysmenorrhea pain starting the same day of the menstruation flow. The majority of the students, 209 (58.7%), had pain onset after menarche from 6 up to 12 months. In about 184 (51.7%), the pain was relieved after 8 h of its onset. The majority of the students 302 (84.83%) had premenstrual syndrome (Table 3).

**Table 3:** Dysmenorrhea pain intensity of female undergraduate students, at Haramaya University, Ethiopia, 2020.

**Characteristics**		**Frequency**	**Percent**
Pain intensity (*n* = 356)	Mild Moderate-severe	111 245	31.2 68.8
Location of the pain (*n* = 356)	Lower abdominal pain Pelvic pain Back pain Groin pain Thigh and leg pain	266 220 289 45 84	74.7 61.8 81.2 12.6 23.6
Pain onset (*n* = 356)	The same day to menstrual flow One day after menstrual flow Two days after menstrual flow 1–2 weeks before flow and other	239 77 13 27	67.1 21.6 3.7 7.6
The pain started after menarche (*n* = 356)	6–12 month 1–2 years 6–2 years Not remember	209 107 22 18	58.7 30.0 6.2 5.1
Free from the pain after onset (*n* = 356)	8 h after 8–72 h after 3 days after and other	184 133 39	51.7 37.3 11.0
Premenstrual syndrome (*n* = 356)	Yes No	302 54	84.83 15.17

### Prevalence of Impact of Dysmenorrhea on Academic Performance

Among students who suffered from dysmenorrhea, 266 (74.7%) reported that the pain adversely affected their academic performance. Difficult to study 245 (92.1%) and difficult to concentrate in class 229 (86.1%) were the most common reported academic impact of students (Table 4).

**Table 4:** Prevalence of impact of dysmenorrhea on academic performance of female undergraduate students, at Haramaya University, Ethiopia, 2020.

**Characteristics**		**Frequency**	**Percent**
Pain impact on academic performance (*n* = 356)	Yes No	266 90	74.7 25.3
Impacts on academic performance (*n* = 266)	Difficult to study Unable to do homework Difficult to concentrate in class Unable to active participation Unable to go to class Unable to do sport No change from other days	245 215 229 146 72 64 25	92.1 80.8 86.1 54.9 27.1 24.1 9.4

### Multivariable Analysis of Factors Associated With the Impact of Dysmenorrhea on Academic Performance

In multivariable analysis, four variables, namely pain intensity, age at menarche, premenstrual syndrome, and pocket money of students were statically significant factors associated with the impact of dysmenorrhea on academic performance. Students who had premenstrual syndromes had an impact of 4.86 times on academic performance than those who had no premenstrual syndromes [AOR = 4.86:95%CI (2.13, 11.06)]. Students who had moderate to severe dysmenorrhea pain intensity had 8.53 times an impact on academic performance [AOR = 8.53:95%CI (4.45, 16.39)] compared with students who had mild pain intensity. Age of menarche ≤ 12 years had an impact of 4.89 times on academic performance compared with the age of menarche ≥15 [AOR = 4.89:95%CI (2.03, 11.77)]. Students who had monthly pocket money <150ETB had an impact of 3.91 times on academic performance than students who had monthly pocket money of 300–6000ETB [AOR = 3.91:95%CI (1.48, 10.29)] (Table 5).

**Table 5:** Multivariable analysis of factors associated with the impact of dysmenorrhea on academic performance among female undergraduate students, at Haramaya University, Ethiopia, 2020.

**Variables**		**Dysmenorrhea impact on academic performance**		**AOR(95%CI)**	***P*-value**
		**Yes**	**No**		
Premenstrual syndrome	Yes	240	62	4.86 (2.13, 11.06)	0.000
	No	26	28	1.00	
Age at menarche	≤ 12 years	107	18	4.89 (2.03,11.77)	0.000
	13–14 years	116	40	2.49 (1.18,5.23)	0.016
	≥15 years	43	32	1.00	
Student's monthly pocket money	<150 ETB	59	10	3.91 (1.48, 10.29)	0.006
	150–300 ETB	70	15	1.76 (0.76, 4.08)	0.18
	>300 ETB	137	65	1.00
Pain intensity	Moderate-Severe	212	33	8.53 (4.45, 16.39)	0.000
	Mild	54	57	1.00

## Discussion

In this study, prevalence and the associated factors of impact of dysmenorrhea on academic performance among undergraduate regular students of Haramaya University were assessed.

This study showed that among students with dysmenorrhea, 74.7% [95%CI: (70.0–79.5%)] reported that dysmenorrhea had an impact on their academic performance. Some of its impacts were as follows: unable to study for an exam (92.1%), loss of class concentration (86.1%), inability to do homework (80.8%), loss of class participation (54.9%), class absence (27.1%), and limited sports participation (24.1%). This prevalence is in line with a previous study conducted in Gondar, Ethiopia, which was 74.1% (23). This prevalence is higher than those in previous studies conducted in Ethiopia 20–62.8% (5, 6, 21) and lower than previous studies conducted in Iraq 79.9% (22) and 88.3% in Ethiopia (10). This variation would be because of the self-reporting nature of the operational definition of academic impacts, university students' various disciplines, and the difference in socio-demographic characteristics of the students.

In this study, those students who had a moderate to severe dysmenorrhea pain intensity were found to have a significant association with the occurrence of the impact of dysmenorrhea on academic performance, which was 8.53 times more likely compared to mild dysmenorrhea pain. This is consistent with reports from other studies (15, 16, 22, 24, 25). This could be due to the following: no standard measure used for dysmenorrhea pain intensity, hormonal change in the luteal phase resulting in mood changes, and other symptoms which interfere with students' daily academic activity. Furthermore, this study showed that early menarche (≤ 12 years) was 4.89 times more likely to cause an impact of dysmenorrhea on academic performance compared to late menarche ≥15 years, which is consistent with the findings of other studies in Nigeria (26). This might be due to early menarche reflecting longer exposure to uterine prostaglandins that play a major role in dysmenorrhea through increasing uterine contractility resulting in pain, and this pain interferes with the students' academic performance.

In this study, students having premenstrual syndrome were found to have a significant association with the occurrence of the impact of dysmenorrhea on academic performance, which was 4.86 times more likely compared to students who had no premenstrual syndromes. This is consistent with the report of another study in Egypt (27). The possible justification for this might be the hormonal changes that result from physical and psychological symptoms that interfere with students' academic activity. Students having monthly pocket money <150 ETB were found to have a significant association with the occurrence of the impact of dysmenorrhea on academic performance, which was 3.91 times more likely compared to students who had monthly pocket money 300–6,000 ETB (10). The possible justification would be those students who had not gotten enough money are exposed to depression than students who have enough money, which results in aggravating painful menstruation that affects their academic performance.

## Limitations of the Study

The cross-sectional nature of this study and dysmenorrhea academic impacts were reported looking-back. All students were from one university might raise the issue of generalization. However, this is a first approach, and based on these findings it would be interesting for future researchers to explore more in longitudinal studies to establish the causal effect of dysmenorrhea on academic performance.

## Conclusions and Recommendations

In this study, there was a high prevalence of the impact of dysmenorrhea on academic performance among undergraduate female students of Haramaya University. The most common impact was difficulty studying, unable to do homework, difficulty concentrating in class, unable to actively participate, unable to go to class, and unable to do sports activities. Dysmenorrhea pain intensity, age at menarche, premenstrual syndrome, and students' monthly pocket money were found to be independent determining factors for the occurrence of the impact of dysmenorrhea on academic performance.

Awareness should be created among the authorities and teachers of Haramaya University about the impact of premenstrual syndrome and dysmenorrhea pain intensity on academic performance to provide psychological and academic guidance, and managing mechanisms for the affected students. Haramaya University has to establish medical care for the affected students.

## Data Availability Statement

The original contributions presented in the study are included in the article/Supplementary Materials, further inquiries can be directed to the corresponding author.

## Ethics Statement

The studies involving human participants were reviewed and approved by Haramaya University College of Health and Medical Sciences Institutional Health Research Ethics Review Committee (HU-IHRERC). The patients/participants provided their written informed consent to participate in this study.

## Author Contributions

TM, HA, AS, and TA were involved in title selection, design of the study, literature search, review, data collection and analysis, data interpretation, and report writing. All authors read and approved the final manuscript.

## Conflict of Interest

The authors declare that the research was conducted in the absence of any commercial or financial relationships that could be construed as a potential conflict of interest.

## Publisher's Note

All claims expressed in this article are solely those of the authors and do not necessarily represent those of their affiliated organizations, or those of the publisher, the editors and the reviewers. Any product that may be evaluated in this article, or claim that may be made by its manufacturer, is not guaranteed or endorsed by the publisher.

## Abbreviations

CMHS: college of health and medical sciences
CSSH: college of social sciences and humanities
CNCS: college of natural and computational sciences
SPSS: statistical packages for social sciences
AOR: adjusted odds ratio
ETB: ethiopian birr
ID: identification card.

## References

[1] Ahuja A Manoj K. Sharma, Amarjeet S. Impact of dysmenorrhea on quality of life of adolescent girls of Chandigarh. J Child Adolesc Behav. (2016) 4:295. 10.4172/2375-4494.1000295

[2] ACOG. Dysmenorrea, Painful Periods: Womens Health Care Physicians. The American College of Obstetricians and Gynecologists (2015).

[3] Chauhan G Kodnani A. A study of prevalence and impact of dysmenorrhea and its associated symptoms among adolescent girls residing in slum areas of Vadodara city, Gujarat. Int J Med Sci Public Health. (2016) 5:510. 10.5455/ijmsph.2016.20102015145

[4] Al-Matouq S Al-Mutairi H Al-Mutairi O Abdulaziz F Al-Basri D Al-Enzi M. Dysmenorrhea among high-school students and its associatefactors in Kuwait. BMC Pediatr. (2019) 19:80. 10.1186/s12887-019-1442-6 30885151 PMC6421654

[5] Azagew AW Kassie DG Walle TA. Prevalence of primary dysmenorrhea, its intensity, impact and associated factors among female students' at Gondar town preparatory school, Northwest Ethiopia. BMC Womens Health. (2020) 20:5. 10.1186/s12905-019-0873-4 31906945 PMC6945628

[6] Derseh B. Prevalence-of-dysmenorrhea-and-its-effects-on-school-performance-a-crosssectional-study. J Women's Health Care. (2017) 6:361. 10.4172/2167-0420.1000361

[7] Oni TH Shitangano TGT. Prevalence of menstrual disorders and its academic impact amongst tshivenda speaking teenagers in rural South Africa. J Hum Ecol. (2015) 51:214–9. 10.1080/09709274.2015.11906915

[8] Tolossa FW Bekele ML. Prevalence, impacts and medical managementsof premenstrual syndrome among female students: cross-sectional study in college of health sciences, Mekelle University, Mekelle, Northern Ethiopia. BMC Women's Health. (2014) 14:52. 10.1186/1472-6874-14-52 24678964 PMC3994244

[9] ACOG. Committee opinion no. 760: dysmenorrhea and endometriosis in the adolescent. Obstet Gynecol. (2018) 132:e249–58. 10.1097/AOG.0000000000002978 30461694

[10] Hailemeskel S Demissie A Assefa N. Primary dysmenorrhea magnitude, associated risk factors, and its effect on academic performance: evidence from female university students in Ethiopia. Int J Womens Health. (2016) 8:489–96. 10.2147/IJWH.S112768 27695366 PMC5034908

[11] Luis B Moazzam A. Recent advances in managing and understanding menstrual disorders. F1000Prime Rep. (2015). 7:33. 10.12703/P7-33 25926984 PMC4371378

[12] Mohammed H Hassen N Musa A. Dysmenorrhea and associated factors among secondary school students in East Hararghe zone, Eastern Ethiopia. East Afr J Health Biomed Sci. (2019) 3:39–48.

[13] WACHS. Dysmenorrea Child and Adolescent Community Health Community Health Manual. Sydney, NSW: Government of Australia (2015). p. 1–6.

[14] Rafique N Al-Sheikh MH. Prevalence of primary dysmenorrhea and its relationship with body mass index. J Obst Gynaecol Res. (2018) 44:1773–8. 10.1111/jog.13697 29974566

[15] Orhan C Çelenay ST Demirtürk F Özgül S Üzelpasaci E Akbayrak T. Effects of menstrual pain on the academic performance and participation in sports and social activities in Turkish university students with primary dysmenorrhea: a case control study. J Obstet Gynaecol Res. (2018) 44:2101–9. 10.1111/jog.13768 30043399

[16] Dahlawi H Bukhari I Alshammari F Althaqib G Alhawasawi M Bashiri R. Impact of dysmenorrheaeffect of dysmenorrhea on the academic performance among students studying in Princess Nourah Bint Abdulrahman University, Riyadh. Int J Med Dev Countries. (2021) 5:588–94. 10.24911/IJMDC.51-1608990432

[17] Gebeyehu MB Mekuria AB Tefera YG Andarge DA Debay YB Bejiga GS. Prevalence, impact, and management practice of dysmenorrhea among university of gondar students, northwestern ethiopia: a cross-sectional study. Int J Reprod Med. (2017) 2017:8. 10.1155/2017/3208276 28589173 PMC5446888

[18] Giletew A Bekele W. Prevalence and associated factors of primary dysmenorrhea among debre tabor university students, North Central Ethiopia. Int J Biomed Eng Clin Sci. (2018) 4:70–4. 10.11648/j.ijbecs.20180

[19] Muluneh AA Nigussie TS Gebreslasie KZ Anteneh KT Kassa ZYl. Prevalence and associated factors of dysmenorrhea among secondary and preparatory school students in Debremarkos town, North-West Ethiopia. BMC Womens Health. (2018) 18:57. 10.1186/s12905-018-0552-x 29699536 PMC5921558

[20] Patel V Tanksale V Sahasrabhojanee M Gupte S Nevrekar P. The burden and determinants of dysmenorrhoea: a population-based survey of 2262 women in Goa, India. BJOG. (2006) 113:453–63. 10.1111/j.1471-0528.2006.00874.x 16489934

[21] Abreu-Sánchez A Ruiz-Castillo J Onieva-Zafra MD Parra-Fernández ML Fernández-Martínez E. Interference and Impact of Dysmenorrhea on the Life of Spanish Nursing Students. Int J Environ Res Public Health. (2020) 17:6473. 10.3390/ijerph17186473 32899505 PMC7559731

[22] Jasim N Rasha A. Dysmenorrhea and its impact on daily activities among secondary school students in Basra, Iraq. J Fac Med Baghdad. (2013) 55:339.

[23] Zegeye D Megabiaw B Mulu A. Age at menarche and the menstrual pattern of secondary school adolescents in northwest Ethiopia. BMC Women's Health. (2009) 9:29. 10.1186/1472-6874-9-29 19804623 PMC2763859

[24] Rodrigues AC Gala S Neves Â Pinto C Meirelles C Frutuoso C. Dysmennorrhea in adolescents and young adults prevalence, related factors and limitations in daily living. Acta Med Port. (2011) 24:383–92. 22849926

[25] Ezebialu I Ezenyeaku C Umeobika J. Prevalence of dysmenorrhea and its contribution to school Absenteeism among Nigerian undergraduate students. Ann Health Res. (2021) 7:59–66. 10.30442/ahr.0701-07-116

[26] Iwuoha EC. Effects of dysmenorrhea on academic and social activities of students in tertiary institutions in an Urban City, South East Nigeria. J Med Princ Clin Pract. (2021) 4:109–15.

[27] Arafa AE Senosy SA Helmy HK Mohamed AA. Prevalence and patterns of dysmenorrhea and premenstrual syndrome among Egyptian girls (12–25 years). Middle East Fertil Soc J. (2018) 23:486–90. 10.1016/j.mefs.2018.01.007

