# Supplementary material for: Impact of Dysmenorrhea on Academic Performance Among Haramaya University Undergraduate Regular Students, Eastern Ethiopia
Source: Front Reprod Health. 2022 Jul 6;4:939035. doi: 10.3389/frph.2022.939035 (PMC9580782; doi:10.3389/frph.2022.939035)
Supplement: Supplementary file 1 [file Table_1.DOCX]

**9.2 Data Collection Instruments**

Code No. -----------

| **Part I:Socio-demographic characterstics of participants** | | |
| --- | --- | --- |
| S.no | Questions | Responses |
| 101 | Age in complete years | …………… |
| 102 | Your average monthly pocket money | ……………… |
| 103 | College | 1.CHMS 2.CSSH 3.CNCS |
| 104 | Acadami c year | 1. 2^nd^ year 2. 3^rd^ and above |
| 105 | Your mother status of education | 1.No formal education 2. Formal education |
| 106 | Do you did physical exercise? | 1. Yes 2. No |
| 107 | Do you have any history of anxiety? | 1. Yes 2. No |
| **Part II: Reproductive characteristics and menstrual pattern of participants** | | |
| 201 | At what age did your first menstration started? | ………………. |
| 202 | Have you ever had sexual intercourse? | 1.Yes 2.No |
| 203 | Is your menstruation cycle comes every 28 days? | 1.Yes 3.No |
| 204 | How many days interval between your menstrual cycle? | ………………..days |
| 205 | For how long days stay your menstruation? | …………………days |
| 206 | How many pads did you changes per a day in your menstruation time? | ………………pads |
| **Part III: Dysmenorrhea pain intensity of the participants** | | |
| 301 | How do you grade the pain severity? | 1.**Mild** (painful menses but seldom inhibt normal daily activity)  2. **Moderate** (daily activity affected and required analgesia which gives relife)  3. **Severe** (daily activity clearly affected and poor effect of analgesia) |
| 302 | In which location the pain occur? (can choose more than one) | 1.Lower abdominal pain/cramping  2.Pelvic pain  3.Back pain  4.Grion pain  5.Thigh pain  6.If others specify……. |

| 303 | When does the pain starts? | 1.The same day on the onset of menstruation  2. a day after the onset of menstruatuion  3. 2 days after the onset of menstruation  4.1-2 weeks before the onset of menstruation  5. If others specify……. |
| --- | --- | --- |
| 304 | When the pain occurs after your first menses started? | 1. After 6-12 months  2. After 1-2 years  3. After 6 months - 2 years  4.If others specify………. |
| 305 | When do you get relieved from the pain after the onset of menstruation? | 1. after 8hrs onset of menstruation  2. after 8-72hrs onset of menstruation  3. after 3 day onset of menstruation  4.if Other specify……. |
| 306 | Do have premenstrual syndrome in your menstruation? | 1. Yes 2. No |
| **Part IV: Prevalence of impact of dysmenorrhea on academic performance** | | |
| 401 | Does the pain have impact on your academic performance? | 1. Yes 2. No |
| 402 | During these painful menstrual periods from which activity of your academic performance you restricted?  (You can choose more than one) | 1. difficult to study  2. unable to do my home works  3. difficult to concentrate in class  4. unable to active participation in class  5. unable to go to class  6. unable to do sport  7. No change from other days |
